# Gender susceptibility to COVID-19: a review of the putative role of sex hormones and X chromosome

**DOI:** 10.1007/s40618-020-01383-6

**Published:** 2020-09-16

**Authors:** C. Foresta, M. S. Rocca, A. Di Nisio

**Affiliations:** Department of Medicine, Unit of Andrology and Medicine of Human Reproduction, University of Padova, Via Giustiniani, 2, 35128 Padua, Italy

**Keywords:** COVID-19, ACE2, TMPRSS2

## Abstract

**Background:**

The recent emergence of COVID-19 poses a global health emergency. One of the most frequently reported data is sex-related severity and mortality: according to the last available analysis on 239,709 patients in Italy, lethality is 17.7% in men and 10.8% in women, with 59% of total deaths being men. Interestingly, the infection rate is lower in males than in females, with 45.8% and 54.2% of positive cases, respectively, suggesting that gender-related factor may worsen disease evolution. A tentative hypothesis to explain these findings is the role of angiotensin-converting enzyme 2 (ACE2) and serine protease TMPRSS2 involved in viral infection.

**Purpose:**

In this review, we summarize the available evidence pointing to gender-related differences in ACE2 and TMPRSS2 expression, from both genetic and endocrine points of view.

**Results:**

Altogether, available evidence points toward two not-mutually exclusive mechanisms in gender susceptibility to COVID-19 by sex hormonal regulation of ACE2 and TMPRSS2. On one hand, ACE2 expression could be increased in women, either by estrogens or constitutively by X chromosome inactivation escape or by reduced methylation, providing a larger reservoir of ACE2 to maintain the fundamental equilibrium of RAS regulatory axis. On the other, low levels of androgens in women may keep at low levels TMPRSS2 expression, representing a further protective factor for the development of COVID-19 infection, despite the increased expression of ACE2, which represents the Trojan horse for SARS-CoV-2 entry.

**Conclusions:**

Both mechanisms consistently point to the role of sex hormones and sex chromosomes in the differential severity and lethality of COVID-19 in men and women.

The recent emergence of the novel, severe acute respiratory syndrome coronavirus (SARS-CoV-2) infection disease (COVID-19) in China and its rapid national and international spread pose a global health emergency. Clinical manifestation of COVID-19 greatly differs by age, sex and other comorbidities. In approximately 20% of cases, the disease evolves into a severe manifestation that requires hospitalization, intubation and may lead to death in up to 10% of patients with other comorbidities. One of the most frequently reported epidemiologic data is sex-related COVID-19 mortality: several studies have reported a significant difference in the rate of severe cases between adult females and adult males (42% vs 58%) [1]. Italy was one of the countries suffering with over 200,000 assessed cases and more than 33,000 deaths [2], with a mortality rate even higher than the one reported in China (14%). According to the last available sex-related analysis from Italian “Istituto Superiore di Sanità” (ISS) on 239,709 patients, lethality in Italy is 17.7% in men and 10.8% in women, with 59% of total deaths being men (Fig. 1). Interestingly, the infection rate is lower in males, with 45.8% and 54.2% of positive cases, respectively [3], suggesting that gender-related factor may worsen disease evolution and symptoms. In China, the death rate for men was 2.8%, compared to 1.7% for women, and males represented 73% of deaths [4, 5]. A recent review on all the available epidemiological studies, collecting data from 59,254 patients from 11 different countries, has shown an association between male sex and higher mortality rate [6]. These findings suggest the presence of a male-related susceptibility. Therefore, being male is as much a risk factor for COVID-19 severity and mortality as being old. Several social factors, genetic, immunological, and hormonal differences, as well as lifestyle habits (i.e., smoking and alcohol consumption, chronic diseases, etc.), have been considered to play a role in this gender disparity. Interestingly, men were also disproportionately affected during the SARS and MERS outbreaks, which were caused by other members of the family *Coronaviridae*. More women than men were infected by SARS in Hong Kong in 2003, but the death rate among men was 50% higher [7]. Although it is assumed that older men have more comorbidities than women of similar age, a tentative hypothesis to explain these epidemiologic findings is the role of the angiotensin-converting enzyme 2 (ACE2): SARS-CoV-2 engages ACE2 in alveolar epithelial cells as the entry receptor and employs the serine protease TMPRSS2 for S protein priming [8, 9]. ACE2 is a crucial component of the renin‐angiotensin system (RAS) (Fig. 1). The classical RAS ACE‐Ang II‐AT1R regulatory axis and the ACE2‐Ang 1‐7‐MasR counter‐regulatory axis play an essential role in maintaining homeostasis in humans. ACE2 is widely distributed in the heart, kidneys, lungs, and testes. ACE2 antagonizes the activation of the classical RAS system and protects against organ damage, protecting against hypertension, diabetes, and cardiovascular disease. Similar to SARS‐CoV, SARS‐CoV‐2 also uses the ACE2 receptor to invade human alveolar epithelial cells. Acute respiratory distress syndrome (ARDS) is a clinical high‐mortality disease, and ACE2 has a protective effect on this type of acute lung injury [10, 11]. Interestingly, also in the infection by H5N1, an increase of AngII levels was observed, and they were positively associated with the severity of the disease. Infected mice also showed a downregulation of ACE2 in lungs and an increase of AngII [12]. So far, the binding of COVID-19 spike protein to ACE2 has been shown to downregulate ACE2 and, in turn, to decrease angiotensin 1–7 production. This mechanism may be involved in the pathogenesis of pulmonary hypertension and insufficiency caused by SARS-CoV-2 infection [13]. Therefore, the downregulation of ACE2 expression in SARS-CoV-2 patients may play a causal role in the pathogenesis of COVID-19, as observed in ARDS, SARS-CoV and H5N1, which provides a reasonable explanation for the progression into ARDS.

**Fig. 1:**
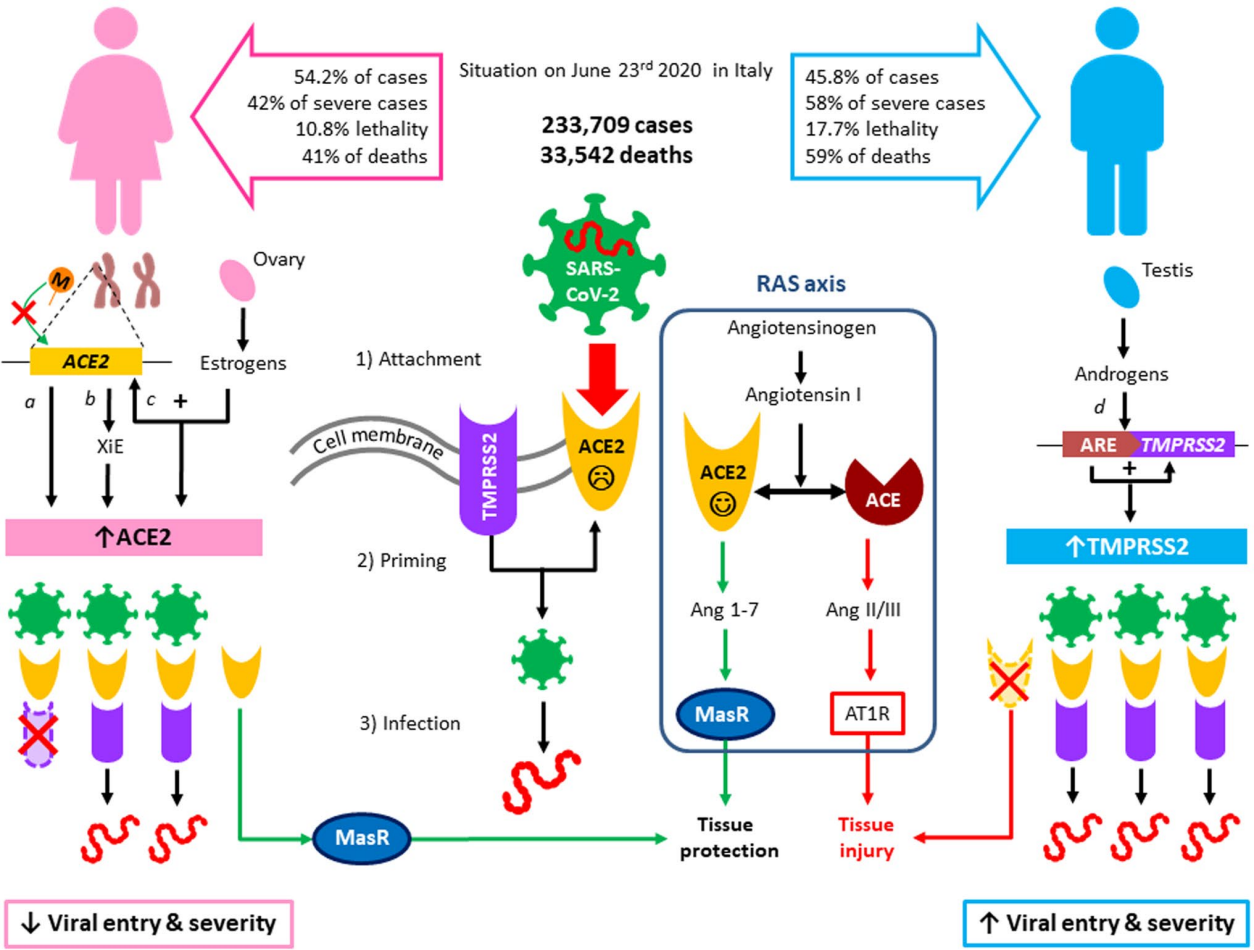
Proposed mechanisms of sex-related susceptibility to COVID-19. On the top of the figure, epidemiological data from the Italian Ministry of Health are reported, with respective gender distribution of cases and deaths. A schematic representation of SARS-CoV-2 mechanism of infection is reported: after binding of viral spike proteins to angiotensin-converting enzyme 2 (ACE2) (1), transmembrane serine protease-2 (TMPRSS2) primes S protein (2), favoring viral entry and infection (3). ACE2 is also crucial in tissue response to viral infection, as it is typically involved in the the renin–angiotensin system (RAS), where it converts angiotensin I into angiotensin 1–7 (Ang 1–7), which binds to Mas receptor (MasR) and favors tissue protection, mainly by hypotensive and anti-inflammatory pathways. Conversely, ACE converts angiotensin I into angiotensin II/III (Ang II/III) that binds to angiotensin II type 1 receptor (AT1R), favoring tissue injury. On the left, the main mechanisms involved in reduced COVID-19 severity and mortality in women are proposed: (a)* ACE2* methylation (M, orange dot) is reduced in women, resulting in higher ACE2 expression; (b) *ACE2* is located on the X chromosome, which in females is present in two copies, in a region of the short arm where 15–30% of genes undergoes X inactivation Escape (XiE); (c) estrogens, produced by the ovary, promote ACE2 expression. Increased levels of ACE2 would provide a larger pool for tissue protection (green arrows) after viral entry. On the right, mechanisms involved in male increased susceptibility: (d) androgens, produced by testes, bind to the androgen receptor and are recognized by androgen-responsive elements (ARE) in the promoter of *TMPRSS2,* leading to increased expression, which in turn favors viral entry in males, whereas low levels of androgens in women may keep at low levels TMPRSS2 expression, representing a further protective factor for the development of COVID-19 infection. The lack of increased ACE2 pool in men due to low estrogens would favor the ACE pathway (red arrows) in the RAS axis, which further promotes tissue injury and disease severity in men, compared with women with the same viral load

*ACE2* gene expression in nasal epithelium increases with age [14], but gender differences are not consistent [15–17]. Data from animal experiments and clinical investigations suggest that components of RAS are markedly affected by sex hormones [17, 18]. In particular, estrogen modifies the local renin–angiotensin system homeostasis and achieves protective effects in atrial myocardium by increasing ACE2 expression [19]. Since ACE2 is widely present in human alveolar epithelial, we could hypothesize hormonal regulation even in lung tissues. It was previously reported that estrogen is protective against SARS infection in mice [20]. Interestingly, among these regulatory regions, there are many estrogen receptor-binding motifs and a few androgen receptor-binding motifs [21]. While ACE2 expression in high in infants and highest in adolescent and decrease dramatically in adult males, transgender males who underwent estrogen therapy (estradiol) and androgen deprivation therapy (spirolactone) for 1 year show significantly higher ACE2 expression level and more ACE2-expressing cells among testis Sertoli cells [21]. It should be noted, similar to the ACE2 expression decline over age, both estrogen and androgen are well known to decrease with age. Therefore, the prognosis of severely ill patients with COVID‐19 may be related to the decrease in ACE2 activity in male patients, particularly those with chronic underlying diseases. SARS‐CoV‐2 infection reduces ACE2 activity and receptor consumption, further exacerbating pathophysiological mechanisms, such as Ang II/ACE regulation imbalance. However, this aspect requires further investigations in lung epithelial tissues from COVID-19 patients of both sexes with different severity of the disease.

Interestingly, the gene encoding ACE2 is located on the X chromosome, which in females is present in two copies. However, although they inherit a maternal X chromosome and a paternal X chromosome, following Lyon theory [22], one of the two X chromosomes is transcriptionally silenced, and such inactivation mechanism occurs randomly during late blastocyst stage. This complex process of silencing is controlled by two noncoding RNA and results in the condensation of one X chromosome into a compact structure, referred to as Barr body [23]. The X-inactivation is a fundamental event to ensure a balanced gene expression between sexes; nonetheless, approximately 15–30% of the genes, most of which localize on the short arm (p) of the X chromosome, can escape the inactivation [24, 25]. Since *ACE2* maps at band p22.2, it is likely that it may escape gene inactivation. This phenomenon could hence explain the observed differences in *ACE2* expression between sexes [26].

In addition to X inactivation escape, gene expression of *ACE2* could be regulated through an epigenetic mechanism consisting in DNA methylation at the cytosine-phosphate-guanine (*CpG*) sites mediated by DNA methyltransferases [27]. Environmental or endogenous factor can affect DNA methylation levels resulting thus in an altered gene expression. Interestingly, a recent study performed on patients with essential hypertension has found a higher *CpG* methylation in patients compared to controls; furthermore, they also reported a statistically significant difference in DNA methylation levels between healthy males and healthy females. The latter finding could, hence, further support a sex-related *ACE2 g*ene expression [28].

In conclusion, ACE2 not only provides a pathway for viral infection, but also represents a protective factor in the severity of the disease. This double-faced role could seem in contrast with epidemiological data. A possible explanation is that the binding of SARS-CoV-2 to ACE2 leads to a rapid saturation of the latter and a disequilibrium of RAS axis in lungs, leading to multi-organ inflammation due to the prevalence of the AT1 pathway. Therefore, under the same viral load in the two sexes, we could hypothesize a more rapid saturation of ACE2 in men than in women, who present a larger pool of ACE2 thanks to hormonal and genetic features, representing a protective factor in the onset of the disease (Fig. 1).

Interestingly, ACE2 is highly expressed also in the testis [29]. On this basis, recent hypotheses suggested that the testis could represent a viral reservoir and may play a role in viral persistence in males. However, this hypothesis is unlikely and, in the only study available to date, SARS-CoV-2 was not detected in testicular biopsies and seminal fluid [30, 31]. Moreover, SARS-CoV-2 could reach the testis only by blood, but to date evidence of serum viral load is still controversial [32–34]. The presence of SARS-CoV-2 in the testis and seminal fluid clearly needs further investigations and could have important clinical implications in our understanding of viral infection and transmission [35]. Data derived from other SARS-CoV infections suggest that in patients recovered from COVID-19, especially for those in reproductive age, andrological consultation and evaluation of gonadal function including semen analysis should be suggested [36].

As previously reported, SARS-CoV-2, as well as other coronaviruses and influenza viruses, critically depends on TMPRSS2 for viral entry and spread in the host [8, 37, 38], highlighting the central and conserved role of TMPRSS2 in the pathogenesis of viral-related diseases. However, the expression of this protein is very low in the testis [39], making viral accumulation in male gonads unlikely.

A more promising hypothesis to explain gender differences of COVID-19 severity rather relies on the role of sex hormones, as previously demonstrated for ACE2. This hypothesis also applies to TMPRSS2, which was first identified in prostate cancer cells, where it is strongly upregulated in response to androgens [40]. In fact, the androgen-responsive element is the only known transcription promoter for the TMPRSS2 gene and this has been strongly associated with the acquired tumor growth and invasiveness of prostate cancer, particularly when TMPRSS2-ERG gene fusion is observed (Reviewed in [41]) and suppressing circulating androgens in men might reduce its activity and reduce the severity of COVID infection [42]. Immunohistochemistry studies, although with limited sample size, suggest that the TMPRSS2 protein is more heavily expressed in bronchial epithelial cells than in surfactant-producing type II alveolar cells and alveolar macrophages, and that there is no expression in type I alveolar cells that form the respiratory surface [43]; however, there is no evidence of sex difference in *TMPSSR2* mRNA levels in the lung [39]. Understanding how TMPRSS2 protein expression in the lung varies could reveal important insights into differential susceptibility to coronavirus infections. Low levels of androgens in women may keep at low levels TMPRSS2 protein expression, representing a further protective factor for the development of COVID-19 infection (Fig. 1). However, it should be recognized that the gap between genders starts being evident after the fifth to sixth decade when, notoriously, serum testosterone declines in men. Accordingly, a recent study has shown that low testosterone represents a predictor of poor prognosis in SARS-CoV-2-infected men [44]. It is tempting to speculate that anti-androgenic therapies used in the treatment of prostate cancer patients might reduce susceptibility to COVID-19 pulmonary symptoms and mortality by reducing testosterone levels and TMPRSS2 activity, but the multifactorial nature of COVID-19 infection and hormonal regulation in men does not allow to draw any definitive conclusion. Given its central role in initiating SARS-CoV-2 infection, factors modulating TMPRSS2 expression or activity could represent a promising candidate for potential interventions against COVID-19 and could add further knowledge on the double-edged role of ACE2 as both an infection-promoting factor and a disease-protective agent.

## Conclusions

Altogether, available evidence points toward two not-mutually exclusive mechanisms in differential gender susceptibility to COVID-19 by sex hormonal regulation of the two main actors in SARS-CoV-2 infection: ACE2 and TMPRSS2. On one hand, ACE2 expression could be increased in women, either by estrogens or constitutively by skewed X chromosome inactivation, providing a larger reservoir of ACE2 to maintain the fundamental equilibrium of RAS-regulatory axis after viral infection on a multi-organ level. On the other hand, low levels of androgens in women may keep at low levels TMPRSS2 expression, representing a further protective factor for the development of COVID-19 infection. Both mechanisms consistently point to the role of sex hormones and chromosomes in the differential severity of SAR-CoV2 infection between sexes, thus representing a one-way avenue to the increased susceptibility to COVID-19 in men. These aspects are worthy of further investigations regarding the epidemiological and biological aspects of this different susceptibility and lethality between sexes. Finally, other gender-related risk factors should be taken into account, such as higher rates of hypertension, smoking, and coronary artery disease that are more predominant in men than women. Altogether, these aspects point toward the man as the true weak sex in COVID-19 challenge.
